# Comparison of surveillance methods applied to a situation of low malaria prevalence at rural sites in The Gambia and Guinea Bissau

**DOI:** 10.1186/1475-2875-8-274

**Published:** 2009-12-02

**Authors:** Judith Satoguina, Brigitte Walther, Christopher Drakeley, Davis Nwakanma, Eniyou C Oriero, Simon Correa, Patrick Corran, David J Conway, Michael Walther

**Affiliations:** 1 Medical Research Council Laboratories, Fajara, PO Box 273 Banjul, The Gambia; 2 Department of Infectious and Tropical Diseases, London School of Hygiene & Tropical Medicine, Keppel Street, London WC1E 7HT, UK; 3 Biotherapeutics Division, National Institute for Biological Standards and Control, Herts EN6 3QG, UK

## Abstract

**Background:**

Health record-based observations from several parts of Africa indicate a major decline in malaria, but up-to-date information on parasite prevalence in West-Africa is sparse. This study aims to provide parasite prevalence data from three sites in the Gambia and Guinea Bissau, respectively, and compares the usefulness of PCR, rapid diagnostic tests (RDT), serology and slide-microscopy for surveillance.

**Methods:**

Cross-sectional surveys in 12 villages at three rural sites were carried out in the Gambia and Guinea Bissau in January/February 2008, shortly following the annual transmission season.

**Results:**

A surprisingly low microscopically detectable parasite prevalence was detected in the Gambia (Farafenni: 10.9%, CI95%: 8.7-13.1%; Basse: 9.0%, CI95%: 7.2-10.8%), and Guinea Bissau (Caio: 4%, CI95%: 2.6-5.4%), with low parasite densities (geometric mean: 104 parasites/μl, CI95%: 76-143/μl). In comparison, PCR detected a more than three times higher proportion of parasite carriers, indicating its usefulness to sensitively identify foci where malaria declines, whereas the RDT had very low sensitivity. Estimates of force of infection using age sero-conversion rates were equivalent to an EIR of approximately 1 infectious bite/person/year, significantly less than previous estimates. The sero-prevalence profiles suggest a gradual decline of malaria transmission, confirming their usefulness in providing information on longer term trends of transmission. A greater variability in parasite prevalence among villages within a site than between sites was observed with all methods. The fact that serology equally captured the inter-village variability, indicates that the observed heterogeneity represents a stable pattern.

**Conclusion:**

PCR and serology may be used as complementary tools to survey malaria in areas of declining malaria prevalence such as the Gambia and Guinea Bissau.

## Background

Although still considered a major international health problem, accumulating evidence indicates that malaria caused by *Plasmodium falciparum *may be on the decline in parts of sub-Saharan Africa. Longitudinal health record-based datasets have recently indicated a significant reduction of the burden of disease in the Gambia [1], in Kenya [2,3] and Eritrea [4] occurring over the last decade.

In February 2008, the Gambian Government launched a policy that malaria should be eliminated as a public health problem. The effectiveness of such efforts needs to be monitored carefully requiring an adequate surveillance system. It has already been recognized that dependent on the aim (control or elimination) and transmission intensity different surveillance methods are likely to be needed [5]. Deriving estimates for prevalence and transmission of malaria from health centre records is unreliable: asymptomatic parasite carriers or cases occurring in communities with difficult access to health care may be missed [6], while common over-diagnosis of malaria [7] results in gross overestimation of the true number of cases, particularly in areas of low transmission [8]. Where elimination is the goal, surveillance should measure the prevalence of the causative agent of the disease directly rather than disease incidence. Thus, sensitive methods to determine parasite prevalence and exposure are required, ideally at the community level. The gold standard for detection of malaria parasites still remains slide microscopy, but it is known for long that a substantial proportion of individuals in a community may have low density infections below the microscopic detection threshold [9]. Such submicroscopic infections contribute substantially to the infectious reservoir [10,11], as they are well capable to infect mosquitoes [12]. Thus, surveillance that aims at identifying the last parasite carrier, requires more sensitive tools such as polymerase chain reaction (PCR) capable to identify as few as 1-10 parasites/μl [13,14]. A recent meta-analysis of studies where parasite prevalence was measured by both PCR and microscopy found that microscopy only detects about 50% of the parasite carriers detected by PCR, and points out that this percentage decreases even further with decreasing transmission [15].

Although surveillance is defined as an ongoing continuous collection of data [16], for practical reasons, monitoring parasite prevalence commonly relies on repeated cross-sectional surveys. Here, the fact that in many areas like the Gambia malaria transmission is highly seasonal constitutes another challenge, as parasite prevalence will vary greatly depending on the timing of data collection. Additional means providing information on exposure over time, ideally allowing the assessment of mid-term trends independent of seasonal variations are highly desirable. In recent years, age-stratified sero-prevalence data of anti-malarial antibodies has been suggested as a useful tool for this purpose [17-19] and may hold particular promise for areas with low malaria transmission: due to the longevity of antibody responses, sero-prevalence data are expected to be higher than parasite rates from cross-sectional surveys and may thus offer a higher sensitivity, and less susceptibility to seasonal fluctuations in transmission. High antibody levels in geographically defined areas may direct targeted control measures and reduction in antibody levels indicate the success of interventions.

Taken together, these considerations indicate the need to employ further tools in addition to or instead of slide microscopy to fully capture parasite prevalence and transmission dynamics at the community level in areas where malaria appears to be on the decline.

The aim of this study was to obtain up to date information on *P. falciparum *malaria parasite prevalence in the Gambia and Guinea Bissau, and to validate the usefulness of PCR, and serology as tools to monitor malaria endemicity in comparison to slide microscopy, and rapid diagnostic tests (RDT). To this end, cross-sectional surveys were carried out in 12 villages at three different sites enrolling a total of 2659 individuals at the end of the malaria transmission in January/February 2008.

## Methods

### Survey procedures

Consecutive cross-sectional surveys were carried out in the Gambia shortly after the end of the malaria transmission season 2008 in the areas around Basse (9^th ^- 15^th ^January), and Farafenni (31^st ^Jan - 7^th ^Feb), and in Guinea Bissau, in the area around Caio (19^th ^- 28^th ^Feb), where the MRC supports a research laboratory. In both countries, malaria transmission is highly seasonal, occurring from August till January, with a peak in November. The study sites have been described in detail elsewhere [20-22]. Based on information from local demographic surveys four villages of appropriate population size were selected at each site that would have at least 45 inhabitants in each of the age groups specified below. After an initial sensitization, those who were willing to participate were grouped according to age, based on the available census data into the following age groups: < 5 years; 5-10; > 11-15; 16-25; 26-40; >40 years. In an attempt to avoid household clustering, all volunteers in each group received consecutive numbers, and the total number of volunteers per group was noted, out of which a random numbers generator drew 45 numbers that identified the participants. Where less than 45 people volunteered per age group, all were enrolled. After written consent was obtained, a questionnaire was administered, approximately 300 μl of blood were obtained by finger prick, and the Hackett score was assessed by a clinician for children aged 1-15 years in all but two villages (S and W). In total, 2,659 people were enrolled (see Additional file 1 for details). The study was reviewed and approved by the Joint Gambian Government/MRC Ethics Committee, as well as the Comité Ético do MINSA, Bissau.

### Assessment of parasite prevalence

Thick blood films were performed in the field and Giemsa-stained in MRC laboratory facilities at Farafenni, Basse and Caio. Slides were examined for the presence of *P. falciparum *malaria parasites by an experienced microscopist, reading 100 high power fields (×1,000) under oil immersion. 10% of slides were read twice as a quality check. An RDT for malaria (OptiMAL) was performed on site, following the manufacturer's instructions. The remaining sample was stored into an EDTA K2 (BD Microtainer Systems, UK) tube. Blood was separated by centrifugation (1,500 rpm, 7 min at RT) into plasma and red blood cells which were subsequently stored separately at -20°C. Upon completion of the survey, DNA was extracted from the pellets using a Corbett Robotics X-Trator Gene™ (Corbett Research Pty Ltd, Australia), and detection of parasite DNA was performed based on nested PCR amplification of the multi-copy 18s rRNA gene as previously described [14]. Positive controls consisting of parasite DNA and a negative control consisting of DNA extracted from malaria-negative blood were included in each PCR run.

### Anti-MSP-1_19 _ELISA

IgG antibody levels binding to MSP-1_19_, (Wellcome allele), fused to glutathione S transferase were measured by indirect ELISA as described [23]. Sera were tested at a single dilution (1:1,000) that has been evaluated previously in the Gambian setting [24].

### Statistics

Taking the age stratified nature of the data into account estimates for the parasite prevalence in the population at each site were calculated as weighted averages of the slide microscopy results in the study population based on the age distribution in each of the villages. To model malaria endemicity based on malarial antibody responses, a reversible catalytic conversion model was fitted to the measured MSP-1_19 _sero-prevalence data from each site stratified into age groups by using standard maximum likelihood methods, based on a binomial error distribution, as described [17,18]. This provides an estimate of the mean annual rate of conversion to seropositive (sero-conversion rate = ⌊), averaged over the age of the population. To exclude the impact of maternally derived antibodies in infants, individuals below 12 month were excluded. To investigate the possibility of sudden changes in transmission (for example due to implementation of highly efficient control measures), further models were fitted in which ⌊ was allowed to change at a single time points during the past 30 years [19]. Likelihood ratio tests against models with fixed ⌊ were used to determine whether there was a significant change in sero-conversion rate. Based on previous estimates [17] a fixed rate of reversion from seropositive to seronegative was fitted (⌊ = 0.0173/year).

To investigate the potential differences in parasite or sero-prevalence within and between sites, separate mixed effect models were fitted for i) parasitaemia assessed by slide microscopy, ii) by PCR, and iii) for anti-MSP-1_19 _antibodies, allowing for the clustering between sites. Initially, variables that were likely to be associated or had already been shown to be associated with malaria prevalence in univariable analysis, such as age, gender, village, ethnicity and bed net use were included in the model. Subsequently, only variables with p-values < 0.05 were kept in the model, which applied to age and gender. In a second step possible additional risk factors such as ethnicity and bed net use were adjusted for.

## Results

### Low malaria prevalence in Gambia and Guinea Bissau

**Figure 1:**
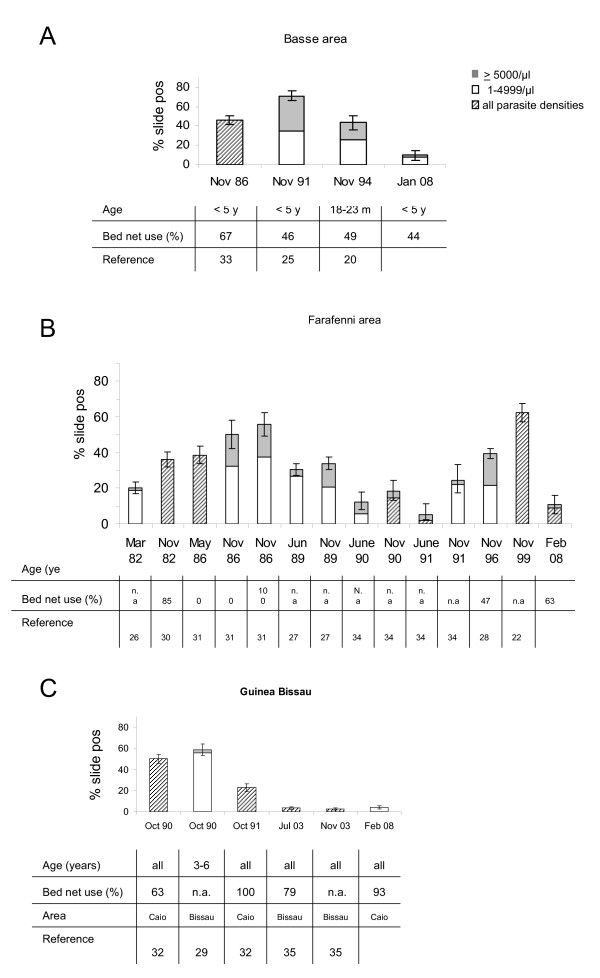
**Current and previous malaria parasite prevalence data from Basse, Farafenni and Caio**. Data from our survey are presented alongside historical data extracted from published reports from A) Basse area, B) Farafenni area or C) Guinea Bissau. Where information on parasite densities was available, percentage of people with 1-4999 and ≥5000 parasites/μl is shown in white and grey blocks, respectively; where parasite densities were not reported, parasitaemia rates are shown as hatched bars. Bars show percentages with 95% CI. Information on age groups surveyed, bed net coverage and the reference are provided in a tabulated format underneath each bar. To enhance comparability our results are displayed for similar age groups as those predominantly reported for this area previously.

The cross-sectional surveys of malaria prevalence described here, carried out in January/February 2008, shortly after the end of the malaria transmission season showed that the parasite prevalence assessed by slide microscopy for those enrolled into the survey was 11.2% (CI 95%. 9.2-13.7%) in Farafenni, 10.4% (CI 95%: 8.6-12.5%) in Basse, and 4% (CI 95%: 2.8-5.5%) in Caio. Taking the age distribution at each village into account, the estimated parasite prevalence in the population in Farafenni, Basse and Caio is 10.9% (CI 95% 8.7-13.1%), 9.0% (CI95%: 7.2-10.8%), and 4% (CI95%: 2.6-5.4%), respectively. Compared to data from similar surveys carried out in the past [20,22,25-34], these are amongst the lowest parasite prevalence data ever reported for Basse and Farafenni. However, considering that malaria transmission in the study area is seasonal, direct comparison to historical data is limited by the fact that surveys were carried out at different times of the year. Whilst the survey presented here was conducted shortly after the end of the transmission season, in Jan/Feb, previously published surveys from Basse [20,25,33] and Farafenni [22,27,28,30,31,34] report data from the peak of the transmission season (Nov), and - in the case of Farafenni - from the middle [26] or the end of the dry season [27,31,34]. However, it is noteworthy that the parasite prevalence measured in Jan/Feb 2008 in the Farafenni area is considerably lower than data obtained in previous years during the dry season (March 82 [26], May 86 [31], June 89 [27], June 90 [34]). In Caio, a rural area of Guinea Bissau, parasite prevalence in Feb 2008 was considerably lower than measured in this area in October 1990 and 1991 [32], and similarly low as described in an urban area of Guinea Bissau in 2003 [35] (Figure 1). Demographic data are presented in Additional file 1.

When parasite prevalence was assessed by qualitative PCR, a three-fold higher percentage (25.5%; CI 95%: 23-7 - 27.3%) of the study population tested positive. The microscopy: PCR prevalence ratio is thus 0.33, indicating that parasite prevalence detected by microscopy is 66% lower than that detectable by PCR.

The RDT detected 8 fold (22 fold) less parasite carriers (1.1% CI 95%: 0.8 - 1.6%) compared to slide microscopy (and PCR; Table 1, 2 and 3). When tested with blood spiked with known concentrations of *P. falciparum *in our laboratory, the detection limit of the RDT was between 140 to 1,400 parasites/μl (D. Nwakanma, unpublished), which is consistent with previous reports indicating considerable loss of sensitivity at levels below 500/μl [36].

**Table 1:** Parasite prevalence detected by PCR

Qualitative PCR				
	**positives**	**Total**	**% positives**	**95% CI**

Basse	219	841	26.0	23.2 - 29.0

Farafenni	185	689	26.9	23.7 - 30.3

Caio	194	818	23.7	20.9 - 26.7

all sites	598	2348	25.5	23.7 - 27.3

**Table 2:** Parasite prevalence detected by RDT

Rapid diagnostic test				
	**positives**	**Total**	**% positives**	**95% CI**

Basse	4	959	0.4	0.2 - 1.1

Farafenni	22	745	3.0	2.0 - 4.4

Caio	2	875	0.2	0.1 - 0.8

all sites	28	2579	1.1	0.8 - 1.6

**Table 3:** Parasite prevalence detected by slide microscopy

Slide microscopy				
	**positives**	**Total**	**% positives**	**95% CI**

Basse	97	934	10.4	8.6-12.5

Farafenni	82	729	11.2	9.2-13.7

Caio	33	834	4	2.8-5.5

all sites	212	2497	8.5	7.5-9.6

Only 14/1,093 children examined had an enlarged spleen, with 57% of them being in village 'T' which had the highest infection prevalence.

### Sero-prevalence of anti MSP-1_19 _antibodies increases continuously with age

**Figure 2:**
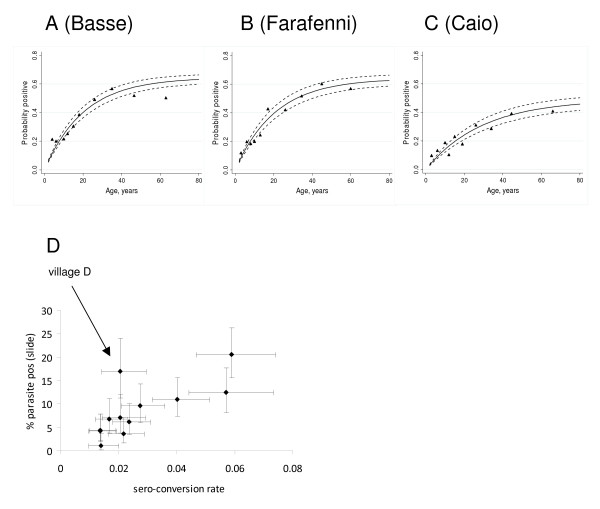
**Continuous increase of MSP-1_19 _antibody sero-prevalence with age**. A-C) For each site the sero-prevalence of anti-MSP-1_19 _antibodies is shown according to age. Triangles represent the observed values, the continuous line shows the values predicted by the catalytic conversion model [17] with 95%

Antibodies against MSP-1_19 _were measured by ELISA and their sero-prevalence plotted according to age for each study site (Figure 2A-C). Sero-prevalence increased at all sites continuously with age. As described in the methods, data from each site were used to fit a model that calculates a fixed sero-conversion rate ⌊(SCR), which is related to the 'force of infection' of malaria [37]. EIR estimates derived from SCR ranged from 0.4 to 4 in the two Gambian sites and 0.2 to 0.5 in Caio. To explore whether sudden changes in transmission (for example due to implementation of highly efficient control measures) have occurred, further models were fitted in which λ was allowed to change at a single time point ("step-model") [19]. However, for all study sites, likelihood ratio tests comparing the step-models to those with fixed ⌊, failed to identify a significant change in sero-conversion rates, suggesting that no sudden decline in transmission has occurred. For one of the villages surveyed in Farafenni samples collected in 1988 were analysed previously using the same ELISA protocol [38]. The estimate for the EIR based on serological parameters [17] was four in 1988, and one in 2008, adding support to the notion of a gradual decline. Subsequently, a model was examined in which the force of infection was allowed to decrease linearly over a 20 -year period. However, the fit was poor suggesting that this simple linear reduction was an oversimplification.

Figure 2D illustrates a significant positive correlation between sero-conversion rate and microscopic parasite prevalence over the 12 villages (Correlation coefficient: 0.73, p = 0.0072). Village D does not fit this trend, as parasite prevalence was relatively high despite a moderate ⌊, which might be explained by very recent malaria exposure that has not yet resulted in increased antibody responses. Removing this value enhances the fit (correlation coefficient: 0.91, p = 0.0001).

### Greater variability in malaria prevalence found within study sites than between

**Figure 3:**
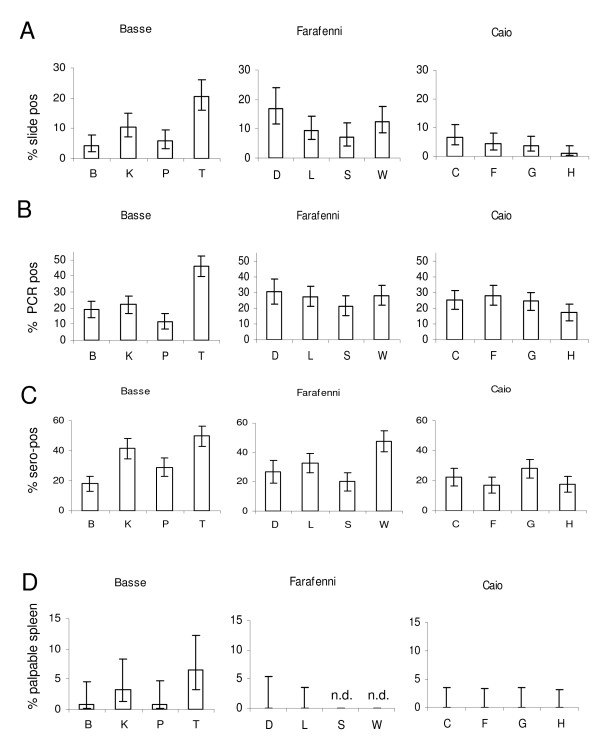
**Variability of parasite -- and sero-prevalence is greatest within study sites**. The prevalence of *P. falciparum *parasitaemia as assessed by A) slide microscopy, or B) by PCR and the C) sero-prevalence of anti MSP-1_19 _antibodies measured by ELISA as well as D) the percentage of children aged 1 to 15 years with a palpable spleen is shown for individual villages at each study site (Basse, Farafenni and Caio). Bars show percentages with 95%

In Figure 3, the prevalence of malaria parasitaemia is shown for each of the surveyed villages as assessed by A) slide microscopy or B) PCR. Data for C) sero-prevalence of anti MSP-1_19 _antibodies and D) spleen rates are displayed in a similar way. For all shown parameters, there is considerable variation between villages within a study site, but less variability between study sites. Using mixed effect models adjusting for age and sex and applied to either slide results, PCR or sero-prevalence data there was no clustering by study site (p = 0.26 [slide], p = 0.21 [PCR], and p = 0.22 [sero-prevalence]), indicating that the variation of malaria prevalence between study sites is not significant.

However, significant inter-village variability was observed for parasite prevalence assessed by slide microscopy in all sites (p < 0.0001 [Basse], p = 0.01 [Farafenni], p = 0.05 [Caio]), and by PCR in Basse (p < 0.0001) and Caio (p = 0.03). Likewise, significant inter-village variability was noted for sero-prevalence in Basse (p < 0.0001) and Farafenni (p < 0.0001). Even after additional adjusting for bed net usage and ethnicity, variability for parasite prevalence within a study site remained significant in Basse (p = 0.001) when assessed by slide reading, and in Basse (p = 0.0002) and Caio (p = 0.02) when assessed by PCR, while the variability for sero-prevalence remained significant in Basse (p < 0.0001) and Farafenni (p < 0.0001). However, since the association of village and malaria prevalence or sero-positivity was highly confounded with ethnicity (see Additional file 1) with some ethnicities (Manjago, Wolof, Serehule) exclusively resident in one site the effect of ethnicity could only be adjusted for partially.

### Peak of parasite prevalence shifts to adolescence

**Figure 4:**
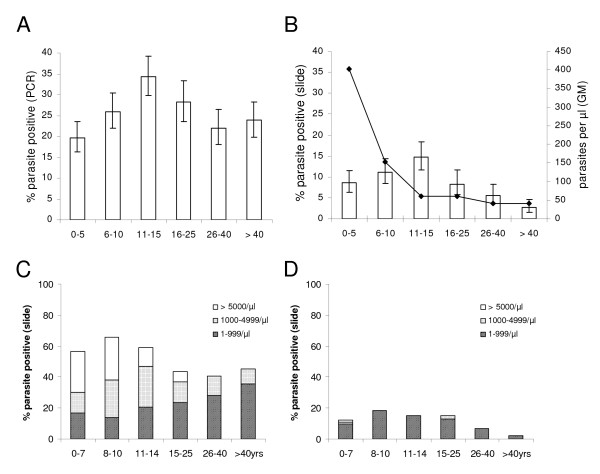
**Peak of parasite prevalence shifts to adolescence**. Parasite prevalence assessed by A) PCR or B) slide microscopy is shown, stratified by age group, showing a significant linear increase from 0-15 years (p < 0.0001 [PCR]; p = 0.003 [slide]). In B) geometric mean parasite densities are added for each age group, showing a significant decline from 0-15 years p < 0.0001), remaining stable thereafter (p = 0.3). Plot C) shows age-stratified parasite prevalence as determined in October 1988 [38] from a village in the Farafenni area. D) Corresponding data from the Farafenni area (villages "D" "L" "S" and "W") from the survey in January 2008 (described in this paper) are shown. Bars show percentages with 95% CI.

Analysis of parasite prevalence detected by PCR (Figure 4A) or slide microscopy (Figure 4B) revealed a significant linear increase with age (test for trend: p < 0.0001 [PCR], p = 0.004 [slide]), peaking in the group of 11-15 years old, followed by a decline in older age groups. Parasite density (4B) dropped significantly with age until adolescence (p < 0.0001) thereafter remaining at low levels (p = 0.3).

For a village at the Farafenni site age-stratified parasite prevalence and density data from October 1988 have been published [38]. Comparison of these data (Figure 4C) to results from the survey in this study (Figure 4D) suggests that the parasite prevalence and parasite densities measured in 2008 are substantially lower than those determined in 1988.

### Bed net use in children under five year of age reduces parasite prevalence measured by slide but not by PCR

**Figure 5:**
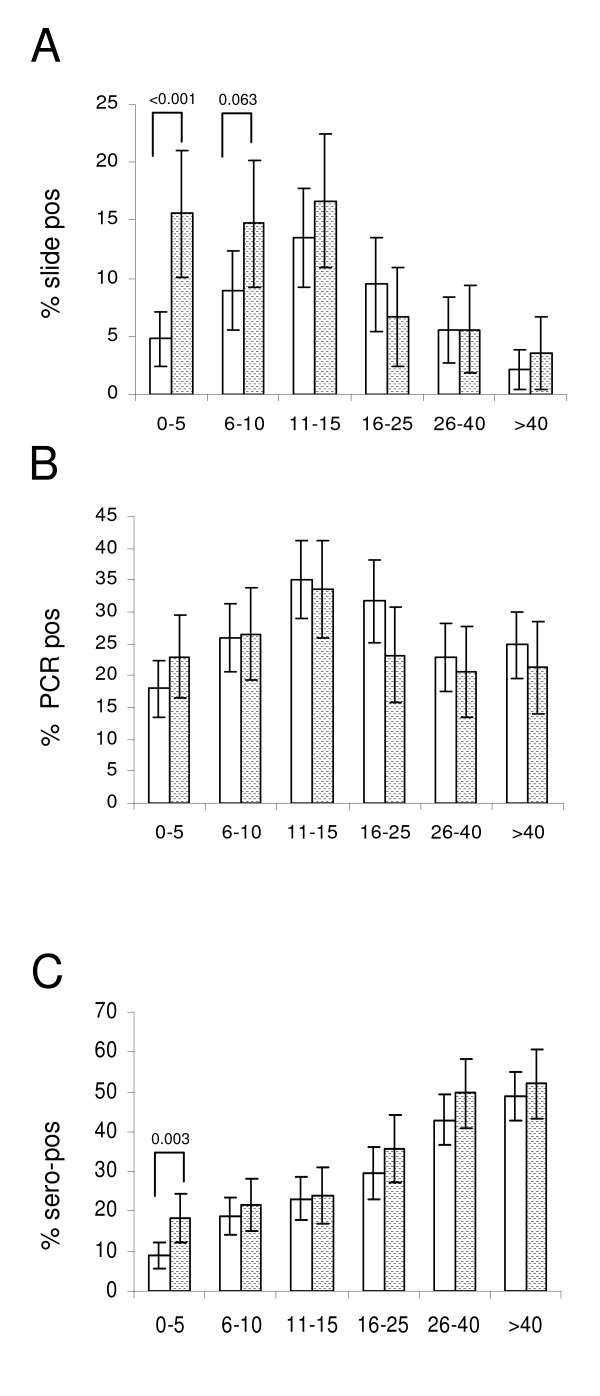
**Bed net use in under 5 year olds reduces parasite prevalence measured by slide but not by PCR**. Age-stratified parasite prevalence assessed by A) slide microscopy or B) PCR, and C) MSP-1_19 _antibody sero-prevalence for individuals sleeping under bed nets (open bars) or not (hatched bars) are shown. Bars represent percentages with 95% CI.

When parasite prevalence assessed by slide microscopy was compared between people sleeping under a bed net (61.9%) and those who didn't, overall bed net-usage had a protective effect with regard to microscopic parasite prevalence (OR 0.63, CI95%: 0.47-0.83, p = 0.001). Especially, children not using a net had a 3.3 fold (0 to 5 years, p < 0.001), and 1.7 fold (6-10 years; p = 0.06) higher parasite prevalence (Figure 5A). For children under the age of five years, the association between bed net use and lower parasite prevalence assessed by microscopy was still significant after adjusting for gender, village, ethnicity and area (OR 0.33, CI95%: 0.16-0.68, p = 0.003), but became non significant in older age groups. This confirms the expected protective effect of bed nets, particularly in the under-five year age group, who belong to the main target population for bed net use. Anti-MSP-1_19 _sero-prevalence for under five year olds sleeping under a bed net was half of that observed for children not using bed nets (p = 0.003, Figure 5C; after adjusting for gender, village, ethnicity and area: OR: 0.53 CI95%: 0.29-0.96, p = 0.035). However, no such differences were observed when PCR-based parasite prevalence data were compared in a similar way (Figure 5B).

## Discussion

The presented surveys revealed surprisingly low levels of microscopically detectable malaria parasite prevalence at all study sites. Although direct comparison to previous studies is considerably limited by differences in study design (e.g. age groups surveyed) and the fact that surveys were carried out at different months of the year (shortly after the end of the transmission season versus peak of transmission season) and in different villages, the parasite prevalence values reported here are amongst the lowest published, remaining below the level reported from surveys carried out at the end of the non-transmission season. The data presented are thus in line with the decline in disease burden documented previously for the Gambia [1]. The renewed efforts to eradicate malaria [39], require surveillance systems that strive to identify the last parasite carriers. The usefulness of PCR, RDT and serology was, therefore, explored as techniques for surveillance.

Not surprisingly, the RDT, having a sensitivity threshold of around 500 parasites/μl, which is designed to detect clinical malaria, proved unsuitable to identify parasite carriers in this low transmission setting, where the geometric mean parasite density, amongst those with microscopically detectable parasitaemia, was as low as 104 parasites/μl (CI 95%: 76 - 143).

Thick film slide microscopy, still considered as the diagnostic gold standard for malaria, missed out 66% of parasite carriers identified by nested PCR that has a detection limit of ~1 to 10 parasites/μl [13,14]. This is in line with a recently published meta-analysis on studies comparing PCR and microscopy for malaria diagnosis in population surveys [15]. This paper further documents, that the proportion of infections missed by microscopy may increase up to 88% where PCR prevalence declines to 10%.

Clearly, the considerable number of individuals with low density parasitaemia missed by microscopy constitutes a major challenge for a malaria surveillance system: given that gametocyte prevalence amongst parasite carriers may be as high as 90% when assessed by Pfs25 QT-NASBA [40], and that even artemisinin-based combination therapy fails to completely abrogate post-treatment infectiousness [40,41], the accurate identification of individuals carrying parasites below the threshold for slide microscopy is necessary to determine the remaining potential for malaria transmission in a community. Here, highly sensitive techniques such as PCR will become indispensable.

The decline in burden of disease documented previously [1], and the reduced parasite prevalence described here occurred after renewed efforts to combat malaria in the Gambia in 2004, such as more efficacious first-line treatment [42], campaigns to retreat bed nets and ITN distribution in the Western Health Region. However, the temporal association between the observed decline and major control interventions does not necessarily prove a causal relationship. In fact, a longitudinal study from Kenya documented a substantial decline in malaria morbidity and mortality even prior to large scale implementation of control measures [3].

To assess whether interventions had a sustained impact, surveillance should therefore be able to capture long and mid-term trends of transmission too, which could be achieved by age profiles of anti -malarial antibody prevalence [17,19]. A successful intervention to control malaria might be expected to result in a distinct "step" in the curve describing the relationship between sero-prevalence and age [18,43], with significantly lower levels of antibodies in individuals born since the introduction of the control measures. To explore whether such a step in the sero-prevalence profile of MSP-1_19 _antibodies was evident, both models with a fixed ⌊ [17] (force of infection), and "step models" where ⌊ was allowed to vary at one point [19] were fitted. For all sites, the models using a single ⌊ gave the best fit, suggesting the decline in transmission is likely to have occurred gradually over several years, and the EIR reduction from 4 to 1 over the last 20 observed here for Farafenni supports this. Further examination on the nature of the gradual decline in transmission using a steady reduction in ⌊ did not provide outcomes with a significantly better fit. Further work is needed to define if the existing data are sufficiently robust to allow fitting a variable force of infection and how the decline in the force of infection is best parameterized.

Considering that the Gambian study sites were outside the areas targeted for ITN distribution in 2004, this is plausible and is supported by Multiple Indicator Cluster Survey (MICS) data available for 2000 [44] and 2005/6 [45] in which questionnaire-based data indicated that the proportion of children < 5 sleeping under an ITN has increased from 13.3% to 44.8% in an area prioritized for ITN distribution (Brikama), whereas it only increased from 14.7% to 16.8% in the Basse area, where parts of this survey were carried out. In areas where malaria transmission is highly seasonal, serology offers the advantage of being considerably less dependent of the timing of the survey than direct assessment of parasite burden by PCR or microscopy that can only provide a snapshot.

Regardless of the method used (slide microscopy, PCR or serology), significant variability of parasite (or sero)-prevalence amongst villages of the same site were observed, whereas the variability between sites did not differ significantly. This remained true even after adjusting for the potentially confounding effects of bed net use and ethnicity, suggesting that other factors may contribute to the variability.

The difference in the distance to malaria breeding sites that has been identified previously as a risk factor for malaria in the Gambia [28], as well as the considerable local variation in both malaria vector density [46] and vector persistence [47] may account for some of the remaining inter-village variability. Serology was equally good at detecting the inter-village variability observed by PCR or microscopy. Considering that sero-prevalence is a cumulative measure of exposure over time and less susceptible to transient fluctuations, the inter-village differences described here may indeed reflect a relatively stable pattern. Where complete data are available (Basse, Caio), this notion is further supported by the distribution of spleen rates in 1-15 year old children (Figure 3D).

In view of the planned malaria elimination efforts that will require adequate surveillance, the spatial heterogeneity of malaria transmission observed locally also constitutes a considerable challenge as it renders the identification of a sentinel village that ought to be representative for surveillance purposes of a larger area difficult. Alternative approaches, such as surveys carried out in schools and/or at health facilities which combine PCR/serology with geographic information (GIS) will become invaluable to identify remaining foci of transmission.

Compared to previous reports, a shift of peak parasite prevalence (both by PCR and slide reading) towards the group of 11-15 years old was observed. This is in sharp contrast to a survey carried out in rural Gambia in the dry season of 1950 where over 95% of children < 5 years and 89% of children aged 6-10 years were found to be parasitaemic [48]. Still in October 1988, parasitaemia rates documented for a village in the Farafenni area remained constantly above 60% until the age of 15 years [38].

While age prevalence profiles of malaria parasitaemia may shift to older age groups with decreasing transmission regardless of its cause, the most obvious reason in our study area probably is the comparably high level of bed net usage in children < 5 years in the Gambia, currently being amongst the countries with the highest coverage of ITNs in children < 5 [49]. As expected, overall bed net-usage had a protective effect with regard to microscopic parasite prevalence. Especially children up to 10 years not using bed nets had significantly higher parasite prevalence. However, no such difference was observed when parasite prevalence was measured by PCR, confirming that bed nets may reduce but not entirely abrogate exposure to malaria. The remaining low level exposure resulting in submicroscopic infections among bed net users may also explain that, with the exception of those < 5 years old, anti-malarial antibodies develop at similar rates regardless whether or not a bed net is used. The notion that anti-malarial immunity can develop despite bed net-usage is consistent with results from longitudinal studies demonstrating that introduction of ITNs [50] or insecticide-treated curtains [51] does not increase child mortality in older children. However, the age shift for peak parasite prevalence also means that acquisition of protective immunity may occur later, which finds its expression in an increased mean age of paediatric malaria admissions [1] - an observation that points out the need to review current guidelines on the management of childhood illness for areas of low malaria prevalence, as these guidelines focus on children below five years [52].

## Conclusion

In summary, the data presented here suggest that particularly in areas of declining malaria prevalence efficient surveillance requires methods able to reliably identify sub-microscopic levels of parasitaemia in order to identify remaining foci of transmission for targeted interventions. Where malaria eradication is the aim the considerable variability in malaria prevalence observed in a confined space calls out for a rather dense network of sentinel sites or alternative approaches. This will require highly sensitive, high throughput techniques such as PCR that show promise even on non-invasive samples [14]. PCR would be well complemented by serological assessment, to adequately capture the current situation on a population level, and also provide information on changes in transmission over time, and a parametric estimation of the entomological inoculation rate [18]. Field samples for both PCR and ELISA can be collected onto filter paper [53], and processed in moderate- to high-throughput assays, where the relevant laboratory equipment is available.

## Competing interests

The authors declare that they have no competing interests.

## Authors' contributions

JS contributed to study design, conducted the fieldwork and the laboratory assays, and contributed to data analysis and drafting the manuscript; BW carried out data management and data analysis; CD analysed serological data, contributed to data interpretation and drafting the ms; DN and EO provided technical expertise and help with the PCR assays; SC helped organize the fieldwork and performed ELISAs; PC provided expertise with the ELISA, and the mathematical modelling; DC helped design the study, and critically revised the ms for important intellectual content; MW conceived, designed and planned the study, helped with the analysis and drafted the ms; All authors have given final approval to the ms.

## Supplementary Material

Additional file 1 Demographic characteristics of the study population at all sites broken down to areas and villages within the areas Click here for file
