# Supplementary material for: Comparison of surveillance methods applied to a situation of low malaria prevalence at rural sites in The Gambia and Guinea Bissau
Source: Malar J. 2009 Dec 2;8:274. doi: 10.1186/1475-2875-8-274 (PMC2791766; doi:10.1186/1475-2875-8-274)
Supplement: Additional file 1 — Demographic characteristics of the study population at all sites broken down to areas and villages within the areas [file 1475-2875-8-274-S1.RTF]

	All Sites	Basse area
	Villages in Basse	Farafenni area	Villages in Farafenni	Caio area	Villages in Caio	
	Total	%	Total	%	B	%	K	%	P	%	T	%	Total	%	D 	%	L	%	S	%	W	%	Total	%	C	%	F	%	G	%	H	%	
N	2,659		983	37.0*	248	25.2#	252	25.7#	234	23.8#	249	25.3#	765	28.8*	152	19.9#	230	30.1#	173	22.6#	210	27.4#	911	34.2*	222	24.4#	224	24.6#	242	26.5#	223	24.5#	
Age																																	
	0-5	510 	19.2	171	17.4	43	17.3	50	19.8	35	15.0	43	17.3	171	22.4	30	19.7	48	20.9	47	27.2	46	21.9	168	18.4	35	15.8	47	21.0	50	20.7	36	16.1	
	6-10	456	17.1	168	17.1	40	16.1	42	16.7	43	18.4	43	17.3	141	18.4	24	15.8	38	16.5	45	26.0	34	16.2	147	16.1	33	14.9	36	16.1	30	12.4	48	21.5	
	11-15	425	16.0	164	16.7	48	19.4	35	13.9	38	16.2	43	17.3	117	15.3	19	12.5	29	12.6	34	19.7	35	16.7	144	15.8	41	18.5	38	17.0	30	12.4	35	15.7	
	16-25	352	13.2	141	14.3	39	15.7	26	10.3	42	17.9	34	13.7	78	10.2	13	8.6	29	12.6	11	6.4	25	11.9	133	14.6	32	14.4	30	13.4	41	16.9	30	13.5	
	26-40	405	15.2	156	15.9	30	12.1	47	18.7	34	14.5	45	18.1	105	13.7	23	15.1	38	16.5	15	8.7	29	13.8	144	15.8	38	17.1	31	13.8	38	15.7	37	16.6	
	>40	431	16.2	159	16.2	42	16.9	39	15.5	39	16.7	39	15.7	133	17.4	34	22.4	44	19.1	18	10.4	37	17.6	139	15.3	33	14.9	35	15.6	44	18.2	27	12.1	
	Unknown	80	3.0	24	2.4	6	2.4	13	5.2	3	1.3	2	0.8	20	2.6	9	5.9	4	1.7	3	1.7	4	1.9	36	4.0	10	4.5	7	3.1	9	3.7	10	4.5	
  Mean age
  (95% CI)	21.4 
(20.6; 22.1)	21.8 
(20.6; 23.0)	21.4
 (19.0; 23.8)	22.0 
(19.5; 24.4)	21.8 
(19.5; 24.2)	21.9 
(19.4; 24.5)	20.5 
(19.2; 21.9)	22.6 
(19.5; 25.6)	22.7 
(20.0; 25.5)	15.7 
(13.3; 18.1)	20.7 
(18.1; 23.3)	21.6 
(20.3; 22.9)	21.7 
(19.2; 24.2)	20.4 
(17.9; 23.0)	23.6 
(20.9; 26.3)	20.4 
(17.9; 22.8)	
Gender																																	
	Male	1,063	40.0	378	38.5	101	40.7	98	38.9	89	38.0	90	26.1	307	40.1	68	44.7	88	38.3	84	48.6	67	31.9	378	41.5	95	42.8	79	35.3	106	43.8	98	44.0	
	Unknown	60	2.3	17	1.7	8	3.2	6	2.4	2	0.9	1	0.4	11	1.4	3	2.0	4	1.7	2	1.2	2	1.0	32	3.5	11	5.0	5	2.2	8	3.3	8	3.6	
Ethnicity																																	
	Fula	138	5.2	115	11.7	16	6.5	2	0.8	94	40.2	3	1.2	13	1.7	-	-	4	1.7	7	4.1	2	1.0	10	1.1	3	1.3	6	2.7	1	0.4	-	-	
	Mandinka	628	23.6	315	32.0	24	9.7	3	1.2	43	18.4	245	98.4	312	40.8	-	-	221	96.1	90	52.0	1	0.4	1	0.1	-	-	1	0.5	-	-	-	-	
	Manjago	830	31.2	-	-	-	-	-	-	-	-	-	-	-	-	-	-	-	-	-	-	-	-	830	91.1	180	81.1	208	92.8	227	93.8	215	96.4	
	Serehule	528	19.9	528	53.7	196	79.0	241	95.6	91	38.9	-	-	-	-	-	-	-	-	-	-	-	-	-	-	-	-	-	-	-	-	-	-	
	Wolof	425	16.0	-	-	-	-	-	-	-	-	-	-	425	55.6	147	96.7	-	-	73	42.2	205	97.6	-	-	-	-	-	-	-	-	-	-	
	Other	44	1.6	7	0.7	5	2.0	-	-	2	0.8	-	-	1	0.1	-	-	-	-	1	0.6	-	-	36	4.0	27	12.2	3	1.3	6	2.5	-	-	
	Unknown	66	2.5	18	1.8	7	2.8	6	2.4	4	1.7	1	0.4	14	1.8	5	3.3	5	2.2	2	1.1	2	1.0	34	3.7	12	5.4	6	2.7	8	3.3	8	3.6	
Bednet use																																	
	YES**	1,645	61.9	401	40.8	53	21.4	63	25.0	100	42.7	185	74.3	422	55.2	36	23.7	213	92.6	109	63.0	64	30.5	822	90.2	196	88.3	183	81.7	231	95.5	212	95.1	
	Unknown	64	2.4	18	1.8	5	2.0	9	3.6	3	1.3	1	0.4	13	1.7	3	2.0	3	1.3	4	2.3	3	1.4	33	3.6	12	5.4	6	2.7	7	2.9	8	3.6	
Supplementary Table 1:  Demographic characteristics of the study population at all sites broken down to areas and villages within the areas


*  	Percent of total study population at all sites (Basse, Farafenni and Caio)
#		Percent of all study participants in area
** 	Slept under a bed net


Demographic details for the study population including information about the distribution of age, gender, ethnicity and bed net use. Village name abbreviations as follows: B: Banico, K: Kumbija,  P: Sara Pirasu, T: Touba Tafsir, D: Dibba Kunda, L: Bambali, S: Sara Kunda, W: Pallen Wollof, C: Binhangai, F: Pupal, G: Tumanbu, H: Caiomete. The column under each area / village-identifier gives the total number of individuals falling into this category. The column to the right expresses this number as a percentage of all study participants of this area/village.
